# Supplementary material for: Clonal Spread of Carbapenem-Resistant Klebsiella pneumoniae Sequence Type 11 in Chinese Pediatric Patients
Source: Microbiol Spectr. 2022 Dec 1;10(6):e01919-22. doi: 10.1128/spectrum.01919-22 (PMC9769831; doi:10.1128/spectrum.01919-22)

Additional file 1 Table S1

General information of ninety-eight *K. pneumoniae* strains

Additional file 2 Table S2

Antimicrobial susceptibilities of ninety-eight *K. pneumoniae* strains

Additional file 3 Figure S1 MLST distribution of strains isolated from different samples and wards.

Different colors show particular K. pneumoniae STs. The circle sizes represent the number of strains.

Additional file 4 Figure S2 Pairwise SNP differences among 98 strains.

Different colors show particular K. pneumoniae STs on the top. Pairwise SNP differences were given by color scale on the right. Pairs of genomes within a distance of 30 SNPs (SNP threshold) are marked by green rectangle.

Additional file 5 Figure S3 Defining the outbreak Clade.

A.Pairs of genomes within a distance of 30 SNPs (SNP threshold) were considered as part of the outbreak.

B. The correlation between the root-to-tip phylogenetic distance (y-axis) and time of sampling (x-axis) within the 62 subclade 1 strains, inferred using TempEst.

Additional file 6 Figure S4 Tajima's D calculated across Clade 1 strains.

Tajima's D values per gene in the Clade 1 genome. Blue dots indicate Tajima's D values for five genes with multiple SNPs.

Additional file 7 Figure S5 Presence/absence matrix of antimicrobial resistance genes and plasmid replicons among all strains.

Core genome phylogeny of 98 strains with different STs are highlighted (left). The colored rectangle (center) indicates the presence of plasmid replicons. The colored rectangle (right) indicates the presence of AMR genes. The classes of resistance genes are also colored on the top.

Additional file 8 Figure S6 CGView Comparison view of two $\boldsymbol{bla_{KPC+}}$ **plasmid.**

CGView Comparison of blast identity between two bla_{KPC}+ plasmids

assembled using long-read data from xz163 and xz164. The *bla*$_{KPC}$+ plasmid from xz163 (outer ring), shares a portion of its genome with the other *bla*$_{KPC}$+ plasmid assembled from xz164 (inner ring;), and might represent a region of recombination between two plasmids circulating in the hospital.

Additional file 9 Figure S7 Plasmids assembled using long-read sequencing.

Schematic representation of seven plasmid assembled using long-read sequencing from the five Clade 1 and four Clade 2 strains. Arrows are proportional to the lengths of the genes and oriented in the direction of transcription. Tracks shown are (from inner to outer): GC skew (G−C/G+ C), G+C content.

Additional file 1 Table S1

Strain	Date	Patient	Gender	Age_days	Samples	Ward	Disease	MLST	*wzi*	K_locus	O_locus
xz091	2018-11-05	Pa32	Female	30	Sputum	Department Of Respiration	Bronchopneumonia	ST3821	wzi374	KL140	O1
xz100	2018-10-14	Pa28	Female	60	Sputum	Gastroenterology	Sepsis	ST76	wzi100	KL10	O3/O3a
xz177	2019-04-26	Pa81	Male	150	Urine	Nephrology	Urinary Tract Infection	ST307	wzi173	KL102	O2
xz068	2018-09-01	Pa15	Male	60	Sputum	PICU	Bronchopneumonia、Sepsis	ST716	wzi89	KL110	O2
xz077	2018-09-21	Pa21	Female	2190	Sputum	PICU	Pneumonia	ST716	wzi89	KL110	O2
xz058	2018-07-21	Pa7	Male	365	Sputum	Department Of Respiration	Bronchitis、ABO Hemolysis	ST11	wzi64	KL64	O2
xz059	2018-07-20	Pa6	Female	30	Sputum	Neonatal Medical Center	Neonatal Pneumonia	ST11	wzi209	KL47	OL101
xz084	2018-10-08	Pa26	Male	365	Blood	Hematology And Oncology	Bacteremia	ST5923	wzi362	KL130	O1
xz061	2018-08-15	Pa9	Female	90	Sputum	CCU	Ventricular Septal Defects	ST11	wzi209	KL47	OL101
xz064	2018-08-17	Pa11	Female	365	Endotracheal Tube	PICU	Sepsis	ST48	wzi62	KL62	O1
xz055	2018-07-10	Pa4	Female	60	Urine	Neonatal Medical Center	Neonatal Respiratory Distress Syndrome	ST76	wzi100	KL10	O3/O3a
xz056	2018-07-13	Pa5	Female	9	Sputum	Neonatal Medical Center	Neonatal Asphyxia	ST76	wzi100	KL10	O3/O3a
xz065	2018-08-25	Pa13	Male	60	Urine	Department Of Respiration	Bronchopneumonia	ST11	wzi209	KL47	OL101
xz066	2018-08-24	Pa12	Male	60	Sputum	CCU	Severe Pneumonia	ST11	wzi209	KL47	OL101
xz060	2018-08-12	Pa8	Male	30	Sputum	Department Of Respiration	Neonatal Sepsis	ST76	wzi100	KL10	O3/O3a
xz069	2018-08-31	Pa14	Male	30	Urine	Urology	Sepsis	ST11	wzi209	KL47	OL101
xz062	2018-08-16	Pa10	Male	120	Blood	SICU	Sepsis、Pneumonia	ST20	wzi82	KL23	O1
xz071	2018-09-05	Pa17	Male	60	Sputum	CCU	Bronchopneumonia	ST11	wzi209	KL47	OL101
xz072	2018-09-10	Pa18	Male	2	Sputum	Neonatal Medical Center	Neonatal Pneumonia	ST11	wzi209	KL47	OL101
xz063	2018-08-18	Pa10	Male	120	Urine	SICU	Sepsis、Pneumonia	ST20	wzi82	KL23	O1
xz074	2018-09-15	Pa20	Male	120	Blood	CCU	Necrotizing Enterocolitis	ST11	wzi209	KL47	OL101
xz070	2018-09-02	Pa16	Female	14	Ascites	Newborn Surgery	Neonatal Pneumonia、Septicemia	ST20	wzi535	KL163	O1
xz079	2018-09-24	Pa22	Male	60	Blood	Department Of Respiration	Bronchopneumonia	ST11	wzi209	KL47	OL101
xz080	2018-09-28	Pa23	Male	30	Sputum	Cardio-Thoracic Surgery	Neonatal Pneumonia	ST11	wzi209	KL47	OL101
xz081	2018-09-30	Pa24	Male	7	Sputum	Neonatal Medical Center	Neonatal Respiratory Distress Syndrome、Neonatal Pneumonia	ST11	wzi209	KL47	OL101
xz083	2018-10-05	Pa25	Male	120	Sputum	Neonatal Medical Center	Bronchitis	ST11	wzi209	KL47	OL101
xz073	2018-09-12	Pa19	Female	90	Sputum	Department Of Respiration	Bronchopneumonia	ST76	wzi100	KL10	O3/O3a
xz087	2018-11-04	Pa31	Female	30	Sputum	CCU	Bronchopneumonia	ST193	wzi275	KL30	O1
xz088	2018-10-11	Pa27	Male	12	Blood	Neonatal Medical Center	Neonatal Sepsis	ST76	wzi100	KL10	O3/O3a
xz090	2018-11-04	Pa30	Male	30	Sputum	Vasculocardiology Deparment	Bronchopneumonia	ST11	wzi209	KL47	OL101
xz099	2019-01-16	Pa53	Female	20	Sputum	NICU	Neonatal Pneumonia	ST193	wzi275	KL30	O1
xz092	2018-11-13	Pa33	Female	24	Ascites	Neonatal Medical Center	Septicemia	ST11	wzi209	KL47	OL101
xz102	2018-10-22	Pa16	Female	60	Blood	Newborn Surgery	Neonatal Pneumonia、Septicemia	ST35	wzi37	KL22	O1
xz105	2018-10-28	Pa29	Female	270	Sputum	CCU	Bronchopneumonia	ST17	wzi386	KL169	OL104
xz122	2018-12-29	Pa43	Male	240	Sputum	Department Of Respiration	Bronchopneumonia	ST76	wzi100	KL10	O3/O3a
xz134	2019-01-12	Pa52	Male	210	Sputum	Department Of Infectious Diseases	Bronchopneumonia	ST76	wzi100	KL10	O3/O3a
xz106	2018-11-17	Pa34	Male	60	Sputum	Vasculocardiology Deparment	Bronchopneumonia	ST11	wzi209	KL47	OL101

Strain	Date	Patient	Gender	Age_days	Samples	Ward	Disease	MLST	*wzi*	K_locus	O_locus
xz107	2018-11-25	Pa35	Male	30	Sputum	PICU	Pneumonia、Septicemia	ST11	wzi209	KL47	OL101
xz051	2018-02-26	Pa1	Female	7	Sputum	NICU	Unknow	ST337	wzi396	KL133	O2
xz109	2018-12-04	Pa37	Male	150	Sputum	PICU	Bronchitis	ST11	wzi209	KL47	OL101
xz110	2018-12-05	Pa38	Male	60	Sputum	PICU	Sepsis、Severe Pneumonia	ST11	wzi209	KL47	OL101
xz112	2018-12-13	Pa37	Male	150	Sputum	Neurology	Bronchopneumonia	ST11	wzi209	KL47	OL101
xz113	2018-12-13	Pa40	Male	30	Blood	PICU	Septicemia	ST11	wzi64	KL64	O2
xz115	2018-12-12	Pa39	Male	150	Sputum	CCU	Bronchopneumonia	ST11	wzi209	KL47	OL101
xz116	2018-12-19	Pa25	Male	180	Sputum	PICU	Severe Pneumonia	ST11	wzi209	KL47	OL101
xz117	2018-12-17	Pa41	Female	210	Sputum	Cardio-Thoracic Surgery	Patent Ductus Arteriosus	ST11	wzi209	KL47	OL101
xz119	2018-12-25	Pa42	Female	120	Sputum	Department Of Respiration	Severe Pneumonia	ST11	wzi209	KL47	OL101
xz121	2018-12-26	Pa25	Male	180	Sputum	PICU	Severe Pneumonia	ST11	wzi209	KL47	OL101
xz052	2018-06-16	Pa2	Male	16	Pus	NICU	Unknow	ST36	wzi27	KL27	O2
xz123	2018-12-31	Pa44	Female	60	Sputum	PICU	Severe Pneumonia	ST11	wzi209	KL47	OL101
xz124	2019-01-05	Pa47	Male	120	Sputum	PICU	Unknow	ST11	wzi209	KL47	OL101
xz125	2019-01-04	Pa6	Female	180	Sputum	CCU	Neonatal Pneumonia	ST11	wzi209	KL47	OL101
xz126	2019-01-04	Pa45	Male	60	Sputum	PICU	Severe Pneumonia、Neonatal Respiratory Distress Syndrome	ST11	wzi209	KL47	OL101
xz127	2019-01-05	Pa46	Female	150	Sputum	Neonatal Medical Center	Unknow	ST11	wzi209	KL47	OL101
xz128	2019-01-05	Pa48	Female	1095	Sputum	PICU	Severe Pneumonia、Sepsis	ST11	wzi209	KL47	OL101
xz053	2018-07-02	Pa3	Female	4745	Blood	Paediatrics	Unknow	ST43	wzi412	KL61	O1
xz131	2019-01-11	Pa51	Female	60	Secretions	Newborn Surgery	Teratoma	ST11	wzi209	KL47	OL101
xz133	2019-01-11	Pa50	Female	150	Secretions	Hematology And Oncology	Severe Pneumonia、Septicemia、Acute Lymphoblastic Leukcmia ALL	ST11	wzi209	KL47	OL101
xz108	2018-11-30	Pa36	Female	30	Sputum	SICU	Pneumonia	ST1140	wzi193	KL125	O3b
xz135	2019-01-02	Pa42	Female	150	Sputum	Department Of Respiration	Severe Pneumonia	ST11	wzi209	KL47	OL101
xz137	2019-01-19	Pa55	Male	22	Sputum	Neonatal Medical Center	Pneumonia	ST11	wzi209	KL47	OL101
xz138	2019-01-19	Pa54	Male	30	Sputum	Newborn Surgery	Bronchopneumonia	ST11	wzi209	KL47	OL101
xz139	2019-01-24	Pa56	Male	180	Sputum	Newborn Surgery	Bronchopneumonia	ST11	wzi209	KL47	OL101
xz140	2019-01-26	Pa57	Male	180	Sputum	Vasculocardiology Deparment	Bronchopneumonia	ST11	wzi209	KL47	OL101
xz141	2019-01-31	Pa59	Female	90	Sputum	Department Of Respiration	Severe Pneumonia	ST11	wzi209	KL47	OL101
xz142	2019-01-31	Pa58	Female	90	Sputum	PICU	Severe Pneumonia	ST11	wzi209	KL47	OL101
xz143	2019-02-04	Pa61	Female	730	Sputum	Cardio-Thoracic Surgery	Down's Syndrome	ST11	wzi209	KL47	OL101
xz144	2019-02-07	Pa62	Male	210	Sputum	Department Of Respiration	Bronchopneumonia	ST11	wzi209	KL47	OL101
xz146	2019-02-27	Pa68	Female	1825	Urine	PICU	Hypoxic-Ischemic Encephalopathy	ST11	wzi209	KL107	OL101
xz147	2019-03-02	Pa69	Male	365	Sputum	CCU	Fallot	ST11	wzi209	KL47	OL101
xz148	2019-03-03	Pa70	Female	60	Sputum	CCU	Severe Pneumonia	ST11	wzi209	KL47	OL101
xz150	2019-03-23	Pa48	Female	1095	Sputum	SICU	Hydrocephalus	ST11	wzi209	KL47	OL101
xz151	2019-03-22	Pa72	Female	2190	Cerebrospinal Fluid	SICU	Central Nervous System Infection	ST11	wzi64	KL64	O2
xz152	2019-03-29	Pa74	Male	4380	Sputum	PICU	Severe Pneumonia	ST11	wzi64	KL64	O2

Strain	Date	Patient	Gender	Age_days	Samples	Ward	Disease	MLST	*wzi*	K_locus	O_locus
xz153	2019-03-28	Pa73	Male	1460	Sputum	SICU	Severe Pneumonia	ST11	wzi64	KL64	O2
xz154	2019-02-07	Pa63	Female	180	Sputum	Department Of Respiration	Severe Pneumonia	ST11	wzi209	KL47	OL101
xz155	2019-02-04	Pa60	Male	90	Sputum	Department Of Infectious Diseases	Pneumonia	ST11	wzi209	KL47	OL101
xz156	2019-02-18	Pa64	Male	300	Sputum	Cardio-Thoracic Surgery	Complete Transposition Of Great Arteries（TGA）	ST11	wzi209	KL47	OL101
xz157	2019-02-18	Pa65	Male	90	Sputum	Department Of Infectious Diseases	Severe Pneumonia	ST11	wzi209	KL47	OL101
xz158	2019-02-21	Pa67	Male	180	Sputum	Newborn Surgery	Acute Bronchitis	ST11	wzi209	KL47	OL101
xz159	2019-02-21	Pa66	Male	180	Sputum	CCU	Unknow	ST11	wzi209	KL47	OL101
xz160	2019-03-04	Pa70	Female	60	Balf	CCU	Severe Pneumonia	ST11	wzi209	KL47	OL101
xz161	2019-03-04	Pa71	Female	120	Sputum	CCU	Pulmonary Hypertension	ST11	wzi209	KL47	OL101
xz163	2019-04-09	Pa74	Male	4380	Sputum	PICU	Severe Pneumonia	ST11	wzi209	KL47	OL101
xz164	2019-04-08	Pa76	Male	365	Sputum	PICU	Severe Pneumonia	ST11	wzi64	KL64	O2
xz166	2019-04-04	Pa75	Male	30	Sputum	Neonatal Medical Center	Neonatal Respiratory Distress Syndrome、Pneumonia	ST11	wzi209	KL47	OL101
xz167	2019-04-14	Pa79	Male	180	Sputum	Department Of Respiration	Bronchopneumonia	ST11	wzi209	KL47	OL101
xz168	2019-04-13	Pa78	Male	90	Sputum	PICU	Sepsis	ST11	wzi209	KL47	OL101
xz169	2019-04-12	Pa77	Male	270	Pus	General Surgery	Intestinal Obstruction	ST11	wzi209	KL47	OL101
xz171	2019-04-19	Pa80	Male	2190	Sputum	PICU	Pneumonia	ST11	wzi64	KL64	O2
xz130	2019-01-09	Pa49	Male	2190	Urine	SICU	Pneumonia	ST1140	wzi193	KL125	O3b
xz178	2019-04-27	Pa82	Male	60	Blood	CCU	Unknow	ST11	wzi209	KL47	OL101
xz179	2019-04-28	Pa83	Male	150	Sputum	Department Of Respiration	Bronchopneumonia	ST11	wzi209	KL47	OL101
xz180	2019-05-07	Pa85	Female	180	Sputum	PICU	Severe Pneumonia	ST11	wzi209	KL47	OL101
xz181	2019-05-03	Pa84	Male	90	Sputum	PICU	Sepsis	ST11	wzi209	KL47	OL101
xz182	2019-05-03	Pa74	Male	4745	Urine	PICU	Severe Pneumonia	ST11	wzi64	KL64	O2
xz183	2019-05-08	Pa86	Male	30	Pus	Newborn Surgery	Atrial Septal Defect	ST11	wzi209	KL47	OL101
xz184	2019-05-21	Pa85	Female	210	Sputum	PICU	Severe Pneumonia	ST11	wzi209	KL47	OL101

Additional file 2 Table S2

id	Strains	Imipenem	Meropenem	Ceftazidime	Cefepime	Aztreonam	Amikacin	Ciprofloxacin	Levofloxacin	Piperacillin/Taxibactam	Cefoperazone/Sulbactam	Polymyxin	Tetacycline	Cotrimoxazole	Gentamicin	Tobramycin
1	xz109	≥16	≥16	≥64	≥64	≥64	≥64	≥4	≥8	≥128	≥64	≤0.5	≤0.5	≤20	≥16	≥16
2	xz125	≥16	≥16	≥64	≥64	≥64	≥64	≥4	≥8	≥128	≥64	≤0.5	≤0.5	≤20	≥16	≥16
3	xz081	≥16	≥16	≥64	≥32	≥64	≥64	≥4	≥8	≥128	≥64	≤0.5	≤0.5	≤20	≥16	≥16
4	xz155	≥16	≥16	≥64	≥64	≥64	≥64	≥4	≥8	≥128	≥64	≤0.5	≤0.5	≤20	≥16	≥16
5	xz143	≥16	≥16	≥64	≥64	≥64	≥64	≥4	≥8	≥128	≥64	≤0.5	≤0.5	≤20	≥16	≥16
6	xz127	≥16	≥16	≥64	≥64	≥64	≥64	≥4	≥8	≥128	≥64	≤0.5	≤0.5	≤20	4	≥16
7	xz074	≥16	≥16	≥64	≥32	≥64	≥64	≥4	≥8	≥128	≥64	≤0.5	≤0.5	≤20	≥16	≥16
8	xz117	≥16	≥16	≥64	≥64	≥64	≤2	≥4	≥8	≥128	≥64	≤0.5	≤0.5	≤20	≤1	≤1
9	xz115	≥16	≥16	≥64	≥64	≥64	≤2	≥4	≥8	≥128	≥64	≤0.5	≤0.5	≤20	≤1	≤1
10	xz066	≥16	≥16	≥64	≥32	≥64	≥64	≥4	≥8	≥128	≥64	≤0.5	≤0.5	≤20	≥16	≥16
11	xz079	≥16	≥16	≥64	≥32	≥64	≥64	≥4	≥8	≥128	≥64	≤0.5	≤0.5	≤20	≥16	≥16
12	xz071	≥16	≥16	≥64	≥32	≥64	≥64	≥4	≥8	≥128	≥64	≤0.5	≤0.5	≤20	≥16	≥16
13	xz141	≥16	≥16	≥64	≥64	≥64	≥64	≥4	≥8	≥128	≥64	≤0.5	≤0.5	≤20	≥16	≥16
14	xz142	≥16	≥16	≥64	≥64	≥64	≥64	≥4	≥8	≥128	≥64	≤0.5	≤0.5	≤20	≥16	≥16
15	xz166	≥16	≥16	≥64	≥64	≥64	≤2	≥4	≥8	≥128	≥64	≤0.5	≤0.5	≤20	≤1	≤1
16	xz112	≥16	≥16	≥64	≥64	≥64	≥64	≥4	≥8	≥128	≥64	≤0.5	≤0.5	≤20	≥16	≥16
17	xz157	≥16	≥16	≥64	≥64	≥64	≤2	≥4	≥8	≥128	≥64	≤0.5	≤0.5	≥320	≤1	≤1
18	xz146	≥16	≥16	≥64	≥64	≥64	≤2	≥4	≥8	≥128	≥64	≤0.5	≤0.5	≤20	≤1	≤1
19	xz135	≥16	≥16	≥64	≥64	≥64	≤2	≥4	≥8	≥128	≥64	≤0.5	≤0.5	≤20	≤1	≤1
20	xz119	≥16	≥16	≥64	≥64	≥64	≤2	≥4	≥8	≥128	≥64	≤0.5	≤0.5	≤20	≤1	≤1
21	xz126	≥16	≥16	≥64	≥64	≥64	≥64	≥4	≥8	≥128	≥64	≤0.5	≤0.5	≤20	≥16	≥16
22	xz092	≥16	≥16	≥64	≥32	≥64	≥64	≥4	≥8	≥128	≥64	≤0.5	≤0.5	≤20	≥16	≥16
23	xz110	≥16	≥16	≥64	≥64	≥64	≥64	≥4	≥8	≥128	≥64	≤0.5	≤0.5	≤20	≥16	≥16
24	xz138	≥16	≥16	≥64	≥64	≥64	≥64	≥4	≥8	≥128	≥64	≤0.5	≤0.5	≤20	≥16	≥16
25	xz178	≥16	≥16	≥64	≥64	≥64	≥64	≥4	≥8	≥128	≥64	≤0.5	1	≤20	≥16	≥16
26	xz123	≥16	≥16	≥64	≥64	≥64	≥64	≥4	≥8	≥128	≥64	≤0.5	≤0.5	≤20	≥16	≥16
27	xz158	≥16	≥16	≥64	≥64	≥64	≥64	≥4	≥8	≥128	≥64	≤0.5	≤0.5	≤20	≥16	≥16
28	xz167	≥16	≥16	≥64	≥64	≥64	≥64	≥4	≥8	≥128	≥64	≤0.5	≤0.5	≤20	≥16	≥16
29	xz083	≥16	≥16	≥64	≥32	≥64	≥64	≥4	≥8	≥128	≥64	≤0.5	≤0.5	≤20	≥16	≥16
30	xz116	≥16	≥16	≥64	≥64	≥64	≥64	≥4	≥8	≥128	≥64	≤0.5	≤0.5	≤20	≥16	≥16
31	xz072	≥16	≥16	≥64	≥32	≥64	≤2	≥4	≥8	≥128	≥64	≤0.5	≤0.5	≤20	≥16	≤1
32	xz121	≥16	≥16	≥64	≥64	≥64	≥64	≥4	≥8	≥128	≥64	≤0.5	≤0.5	≤20	≥16	≥16
33	xz133	≥16	≥16	≥64	≥64	≥64	≥64	≥4	≥8	≥128	≥64	≤0.5	≤0.5	≤20	≥16	≥16
34	xz150	≥16	≥16	≥64	≥64	≥64	≥64	≥4	≥8	≥128	≥64	≤0.5	≤0.5	≤20	≥16	≥16
35	xz131	≥16	≥16	≥64	≥64	≥64	≥64	≥4	≥8	≥128	≥64	≤0.5	≤0.5	≤20	≥16	≥16
36	xz139	≥16	≥16	≥64	≥64	≥64	≥64	≥4	≥8	≥128	≥64	≤0.5	≤0.5	≤20	≥16	≥16
37	xz107	≥16	≥16	≥64	≥64	≥64	≥64	≥4	≥8	≥128	≥64	≤0.5	≤0.5	≤20	≥16	≥16
38	xz090	≥16	≥16	≥64	≥32	≥64	≥64	≥4	≥8	≥128	≥64	≤0.5	≤0.5	≤20	≥16	≥16

id	Strains	Imipenem	Meropenem	Ceftazidime	Cefepime	Aztreonam	Amikacin	Ciprofloxacin	Levofloxacin	Piperacillin/Taxibactam	Cefoperazone/Sulbactam	Polymyxin	Tetacycline	Cotrimoxazole	Gentamicin	Tobramycin
39	xz163	⩾16	⩾16	⩾64	⩾64	⩾64	⩾64	⩾4	⩾8	⩾128	⩾64	⩽0.5	⩽0.5	⩾320	⩾16	⩾16
40	xz168	⩾16	⩾16	⩾64	⩾64	⩾64	⩾64	⩾4	⩾8	⩾128	⩾64	⩽0.5	⩽0.5	⩽20	⩾16	⩾16
41	xz181	⩾16	⩾16	⩾64	⩾64	⩾64	⩾64	⩾4	⩾8	⩾128	⩾64	⩽0.5	⩽0.5	⩽20	⩾16	⩾16
42	xz061	⩾16	⩾16	⩾64	⩾32	⩾64	⩾64	⩾4	⩾8	⩾128	⩾64	⩽0.5	⩽0.5	⩽20	⩾16	⩾16
43	xz128	⩾16	⩾16	⩾64	⩾64	⩾64	⩾64	⩾4	⩾8	⩾128	⩾64	⩽0.5	⩽0.5	⩽20	⩾16	⩾16
44	xz059	⩾16	⩾16	⩾64	⩾32	⩾64	⩾64	⩾4	⩾8	⩾128	⩾64	⩽0.5	⩽0.5	⩽20	⩾16	⩾16
45	xz065	⩾16	⩾16	⩾64	⩾32	⩾64	⩾64	⩾4	⩾8	⩾128	⩾64	⩽0.5	⩽0.5	⩽20	⩾16	⩾16
46	xz124	⩾16	⩾16	⩾64	⩾64	⩾64	⩾64	⩾4	⩾8	⩾128	⩾64	⩽0.5	⩽0.5	⩽20	⩾16	⩾16
47	xz106	⩾16	⩾16	⩾64	⩾64	⩾64	⩾64	⩾4	⩾8	⩾128	⩾64	⩽0.5	⩽0.5	⩽20	⩾16	⩾16
48	xz148	⩾16	⩾16	⩾64	⩾64	⩾64	⩽2	0.5	4	⩾128	⩾64	⩽0.5	⩽0.5	⩽20	⩽1	⩽1
49	xz160	⩾16	⩾16	⩾64	⩾64	⩾64	⩽2	⩾4	⩾8	⩾128	⩾64	⩽0.5	⩽0.5	⩽20	⩽1	⩽1
50	xz183	⩾16	⩾16	⩾64	⩾64	⩾64	⩾64	⩾4	⩾8	⩾128	⩾64	⩽0.5	1	⩽20	⩾16	⩾16
51	xz140	⩾16	⩾16	⩾64	⩾64	⩾64	⩾64	⩾4	⩾8	⩾128	⩾64	⩽0.5	⩽0.5	⩽20	⩾16	⩾16
52	xz147	⩾16	⩾16	⩾64	⩾64	⩾64	⩾4	⩾4	⩾8	⩾128	⩾64	⩽0.5	⩽0.5	⩽20	⩾16	⩾16
53	xz156	⩾16	⩾16	⩾64	⩾64	⩾64	⩾64	⩾4	⩾8	⩾128	⩾64	⩽0.5	⩽0.5	⩽20	⩾16	⩾16
54	xz161	⩾16	⩾16	⩾64	⩾64	⩾64	⩾64	⩾4	⩾8	⩾128	⩾64	⩽0.5	⩽0.5	⩽20	⩾16	⩾16
55	xz159	⩾16	⩾16	⩾64	⩾64	⩾64	⩾64	⩾4	⩾8	⩾128	⩾64	⩽0.5	⩽0.5	⩽20	⩽1	⩾16
56	xz069	⩾16	⩾16	⩾64	⩾32	⩾64	⩽2	⩾4	⩾8	⩾128	⩾64	⩽0.5	⩽0.5	⩽20	⩽1	⩽1
57	xz154	⩾16	⩾16	⩾64	⩾64	⩾64	⩽2	⩾4	⩾8	⩾128	⩾64	⩽0.5	⩽0.5	⩽20	⩽1	⩽1
58	xz179	⩾16	⩾16	⩾64	4	⩾64	⩾64	⩾4	⩾8	⩾128	⩾64	⩽0.5	1	⩽20	⩾16	⩾16
59	xz137	⩾16	⩾16	⩾64	⩾64	⩾64	⩾64	⩾4	⩾8	⩾128	⩾64	⩽0.5	⩽0.5	⩽20	⩾16	⩾16
60	xz169	⩾16	⩾16	⩾32	⩾64	⩾64	⩾64	⩾4	⩾8	⩾128	⩾64	⩽0.5	⩽0.5	⩽20	⩾16	⩾16
61	xz080	⩾16	⩾16	⩾64	⩾32	⩾64	⩾64	⩾4	⩾8	⩾128	⩾64	⩽0.5	⩽0.5	⩽20	⩾16	⩾16
62	xz144	⩾16	⩾16	⩾64	⩾64	⩾64	⩽2	⩾4	⩾8	⩾128	⩾64	⩽0.5	⩽0.5	⩽20	⩽1	⩽1
63	xz184	⩾16	⩾16	⩾64	⩾64	⩾64	⩾64	⩾4	⩾8	⩾128	⩾64	⩽0.5	1	⩽20	⩾16	⩾16
64	xz180	⩾16	⩾16	⩾64	⩾64	⩾64	⩾64	⩾4	⩾8	⩾128	⩾64	⩽0.5	2	⩽20	⩾16	⩾16
65	xz152	⩾16	⩾16	⩾64	⩾64	⩾64	⩾64	⩾4	⩾8	⩾128	⩾64	⩽0.5	⩽0.5	⩾320	⩾16	⩾16
66	xz171	⩾16	⩾16	⩾64	⩾64	⩾64	⩾64	⩾4	⩾8	⩾128	⩾64	⩽0.5	⩽0.5	⩾320	⩾16	⩾16
67	xz164	⩾16	⩾16	⩾64	⩾64	⩾64	⩾64	⩾4	⩾8	⩾128	⩾64	⩽0.5	⩽0.5	⩽20	⩾16	⩾16
68	xz182	⩾16	⩾16	⩾64	⩾64	⩾64	⩽2	⩾4	⩾8	⩾128	⩾64	⩽0.5	4	⩾320	⩽1	⩽1
69	xz058	⩾16	⩾16	⩾64	⩾32	⩾64	⩽2	⩾4	⩾8	⩾128	⩾64	⩽0.5	⩽0.5	⩽20	⩽1	⩽1
70	xz151	⩾16	⩾16	⩾64	⩾32	⩾64	⩾64	⩾4	⩾8	⩾128	⩾64	⩾16	⩽0.5	⩾320	NA	⩾16
71	xz153	⩾16	⩾16	⩾64	⩾64	⩾64	NA	⩾4	⩾8	⩾128	⩾64	⩽0.5	⩽0.5	⩾320	⩽1	8
72	xz113	⩾16	⩾16	⩾64	⩾64	⩾64	⩽2	⩾4	⩾8	⩾128	⩾64	⩽0.5	⩽0.5	⩽20	⩽1	⩽1
73	xz056	⩾16	⩾16	⩾64	⩾32	⩾64	⩽2	⩽0.25	⩽0.25	⩾128	⩾64	⩽0.5	⩽0.5	⩽20	⩽1	⩽1
74	xz055	⩾16	⩾16	⩾64	⩾32	⩾64	⩽2	⩽0.25	⩽0.25	⩾128	⩾64	⩽0.5	⩽0.5	⩽20	⩽1	⩽1
75	xz122	⩾16	⩾16	⩾64	⩾64	⩾64	⩽2	⩽0.25	⩽0.25	⩾128	⩾64	⩽0.5	⩽0.5	⩽20	⩽1	⩽1
76	xz100	8	⩾16	⩾64	⩾64	⩾64	NA	1	1	⩾128	⩾64	⩽0.5	⩽0.5	⩾320	⩽1	8

id	Strains	Imipenem	Meropenem	Ceftazidime	Cefepime	Aztreonam	Amikacin	Ciprofloxacin	Levofloxacin	Piperacillin/Taxibactam	Cefoperazone/Sulbactam	Polymyxin	Tetacycline	Cotrimoxazole	Gentamicin	Tobramycin
77	xz088	⩾16	⩾16	⩾64	⩾32	⩾64	⩽2	⩽0.25	⩽0.12	⩾128	⩾64	⩽0.5	⩽0.5	⩽20	⩽1	⩽1
78	xz073	⩾16	⩾16	⩾64	⩾32	⩾64	⩽2	⩽0.25	⩽0.12	⩾128	⩾64	⩽0.5	⩽0.5	⩽20	⩽1	⩽1
79	xz060	⩾16	⩾16	⩾64	⩾32	⩾64	⩽2	⩽0.25	⩽0.25	⩾128	⩾64	⩽0.5	⩽0.5	⩽20	⩽1	⩽1
80	xz134	⩾16	⩾16	⩾64	⩾32	16	⩽2	⩽0.25	⩽0.25	64	⩾64	⩽0.5	⩽0.5	⩽20	⩽1	⩽1
81	xz091	NA	NA	NA	NA	NA	NA	NA	NA	NA	NA	NA	NA	NA	⩽1	NA
82	xz052	8	⩾16	⩾64	⩾32	⩾64	⩽2	⩽0.25	1	⩾128	⩾64	⩽0.5	⩽0.5	⩽20	NA	NA
83	xz064	⩾16	⩾16	⩾64	⩾32	⩾64	⩽2	⩾4	1	⩾128	⩾64	⩽0.5	⩽0.5	⩾320	⩽1	8
84	xz130	⩾16	⩾16	⩾64	⩾64	⩾64	⩽2	⩽0.25	1	⩾128	⩾64	⩽0.5	⩽0.5	⩽20	⩽1	⩽1
85	xz108	⩾16	⩾16	⩾64	⩾64	⩾64	⩽2	⩽0.25	1	⩾128	⩾64	⩽0.5	⩽0.5	⩽20	⩽1	⩽1
86	xz051	⩾16	⩾16	⩾64	⩾32	⩾64	⩽2	0.5	1	⩾128	⩾64	⩽0.5	2	⩽20	NA	NA
87	xz077	8	⩾16	⩾64	⩾32	4	⩽2	⩾4	⩾8	⩾128	⩾64	⩽0.5	⩽0.5	40	⩾16	2
88	xz068	8	⩾16	⩾64	⩾32	⩽1	⩽2	1	1	⩾128	⩾64	⩽0.5	⩽0.5	⩽20	⩾16	8
89	xz084	⩾16	⩾16	⩾64	⩾32	⩾64	8	⩾4	⩾8	⩾128	⩾64	⩽0.5	⩽0.5	⩾320	⩽1	⩾16
90	xz102	8	⩾16	⩾64	⩾64	16	⩽2	0.5	1	64	⩾64	⩽0.5	⩽0.5	⩽20	⩽1	⩽1
91	xz053	⩾16	⩾16	⩾64	⩾32	⩽1	⩽2	⩽0.25	⩽0.12	⩾128	⩾64	⩽0.5	2	⩽20	NA	NA
92	xz099	⩾16	⩾16	⩾64	⩾64	32	⩽2	⩽0.25	⩽0.25	⩾128	⩾32	⩽0.5	⩽0.5	⩽20	NA	⩽1
93	xz087	⩾16	⩾16	⩾64	⩾32	16	⩽2	0.5	1	⩾128	⩾64	⩽0.5	⩽0.5	⩾320	⩾16	4
94	xz063	⩾16	⩾16	⩾64	⩾32	⩾64	⩽2	⩾4	4	⩾128	⩾64	⩽0.5	⩽0.5	⩾320	⩽1	⩽1
95	xz062	8	⩾16	⩾64	⩾32	⩾64	⩽2	2	4	⩾128	⩾64	⩽0.5	⩽0.5	⩾320	⩽1	⩽1
96	xz070	⩾16	⩾16	⩾64	⩾32	⩾64	⩽2	⩾4	NA	NA	⩾64	⩽0.5	⩽0.5	⩽20	⩾16	NA
97	xz177	8	⩾16	⩾64	⩾64	⩽1	⩽2	⩾4	⩾8	8	⩾64	⩽0.5	⩽0.5	⩽20	⩽1	⩽1
98	xz105	8	⩾16	⩾64	⩾64	16	⩽2	0.5	1	⩾128	⩾64	⩽0.5	⩽0.5	⩾320	⩾16	2

Additional file 3 Figure S1

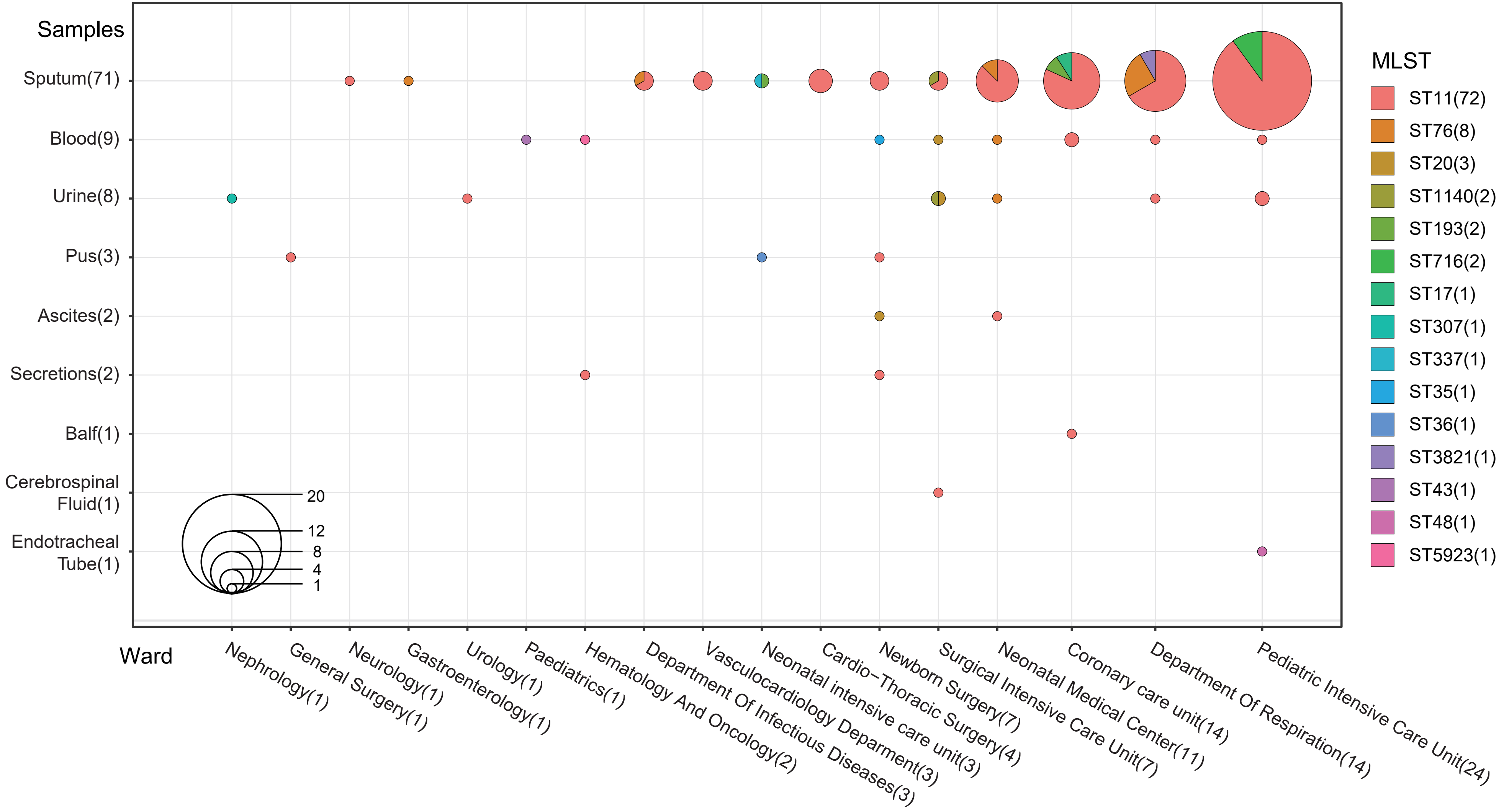

Additional file 4 Figure S2

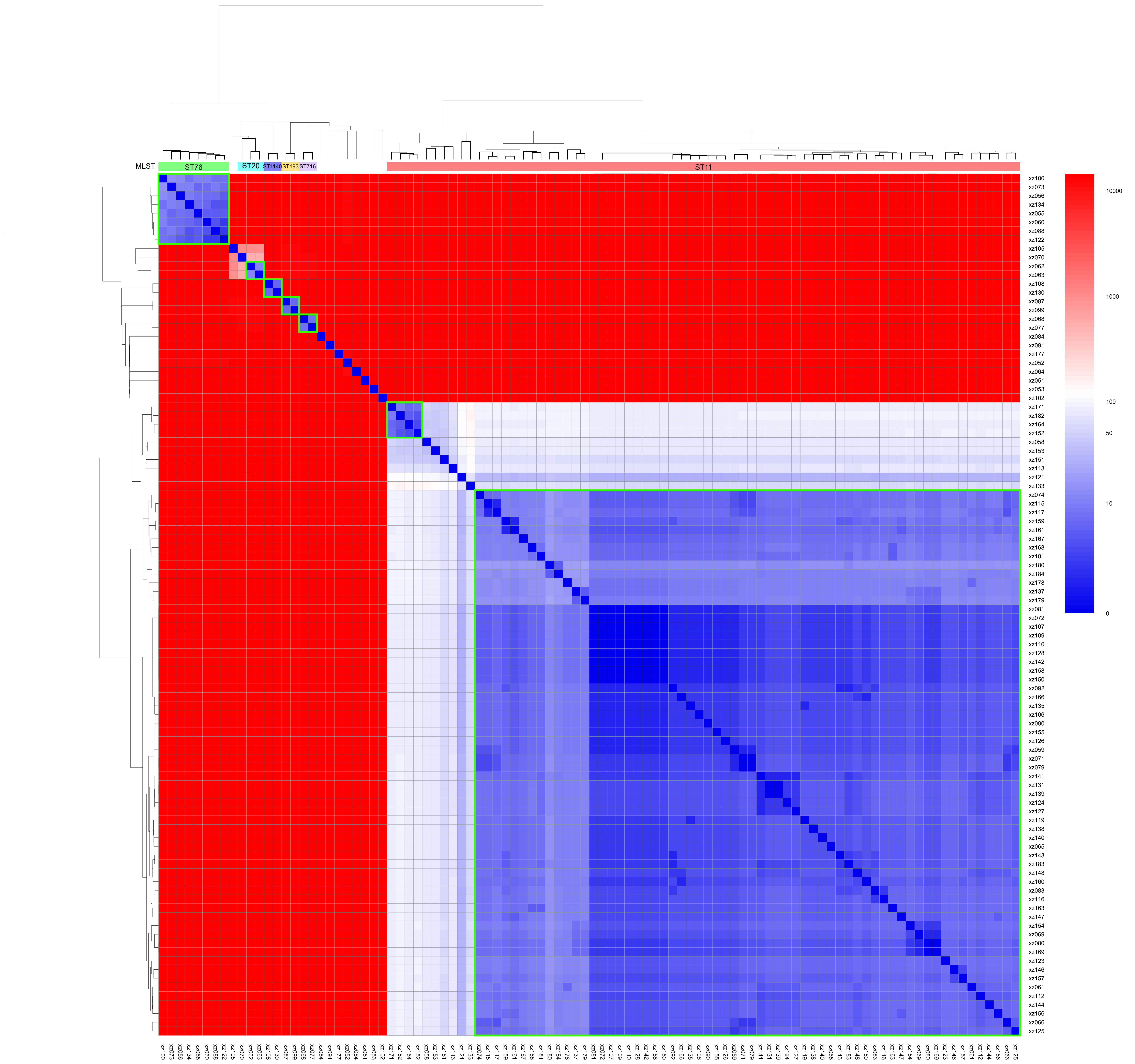

Additional file 5 Figure S3

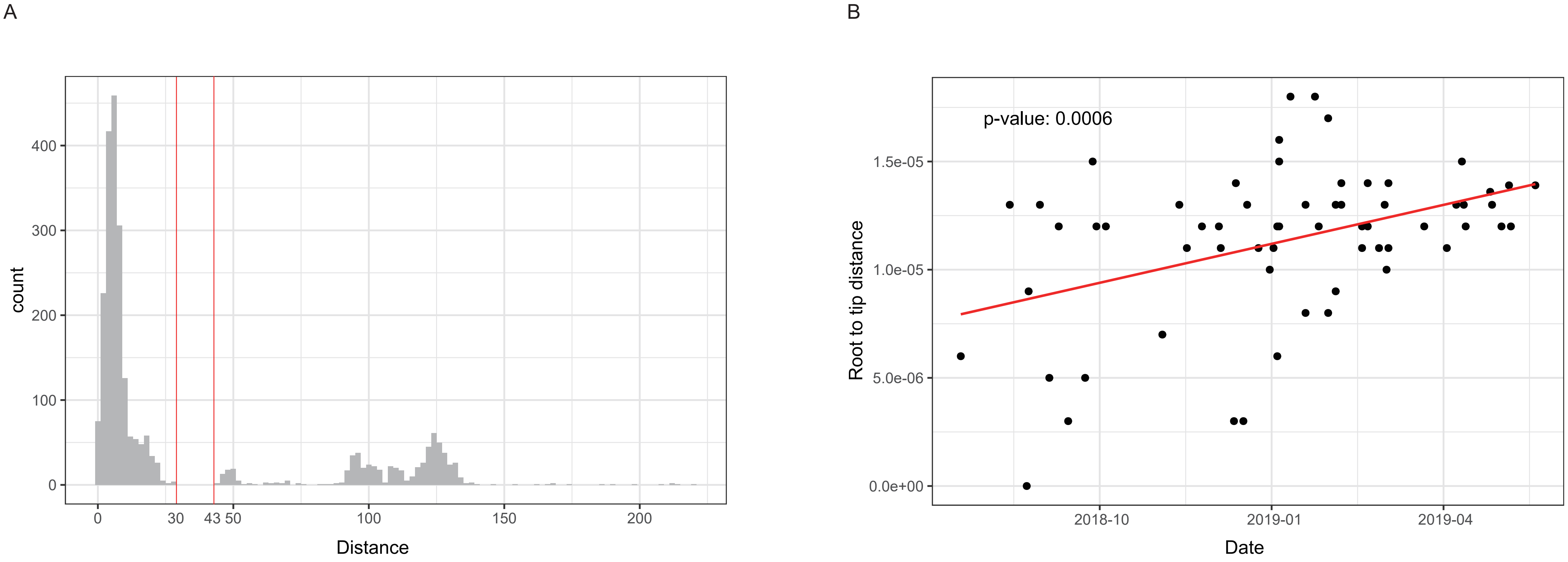

Additional file 6 Figure S4

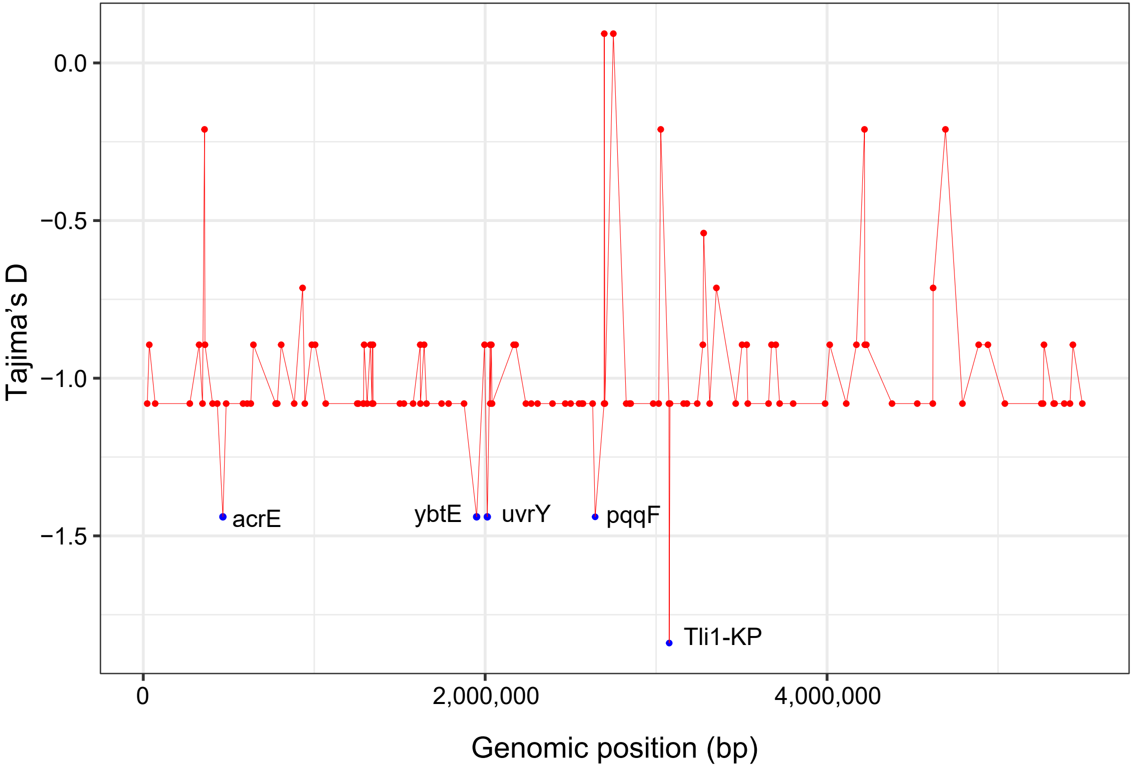

Additional file 7 Figure S5

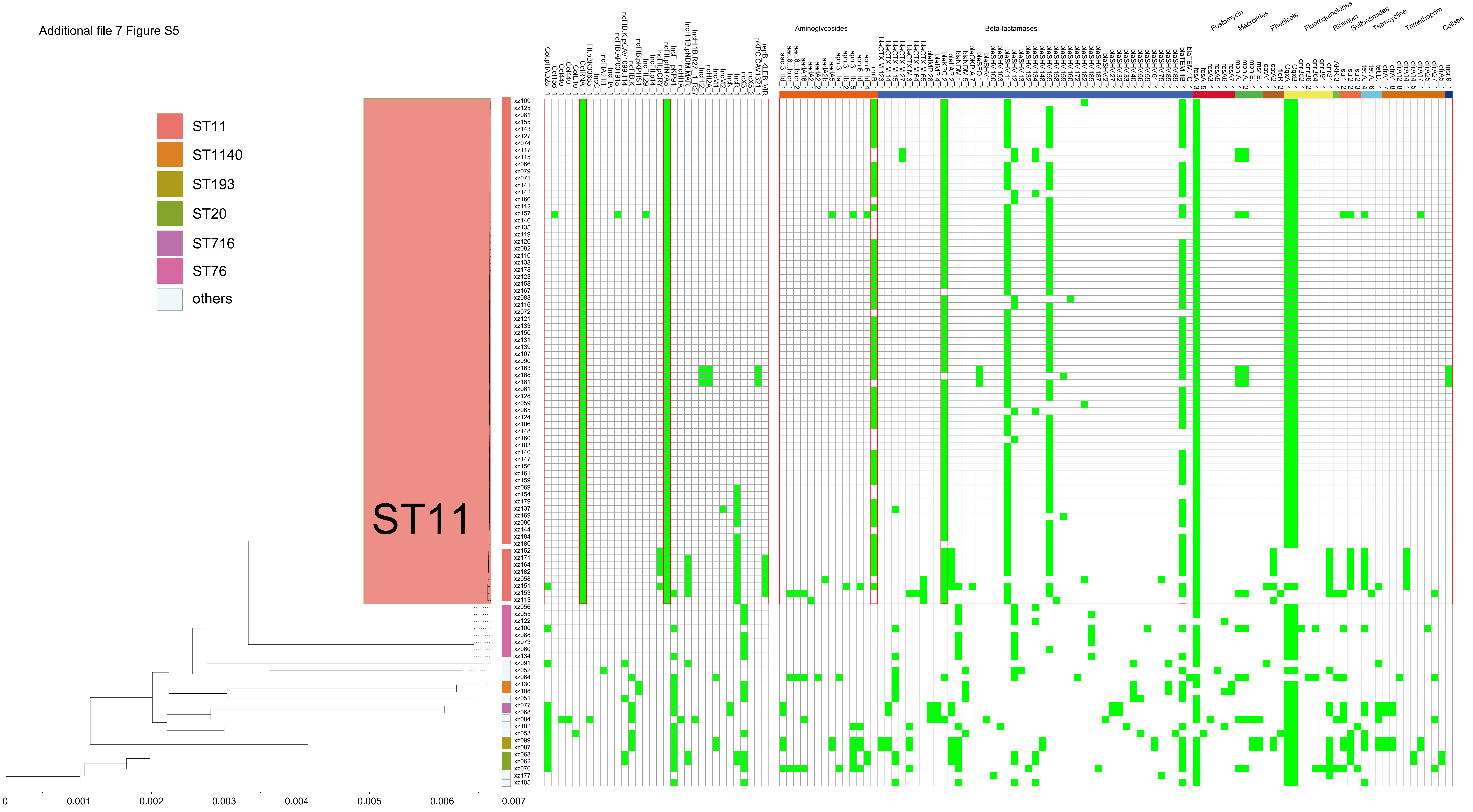

Additional file 8 Figure S6

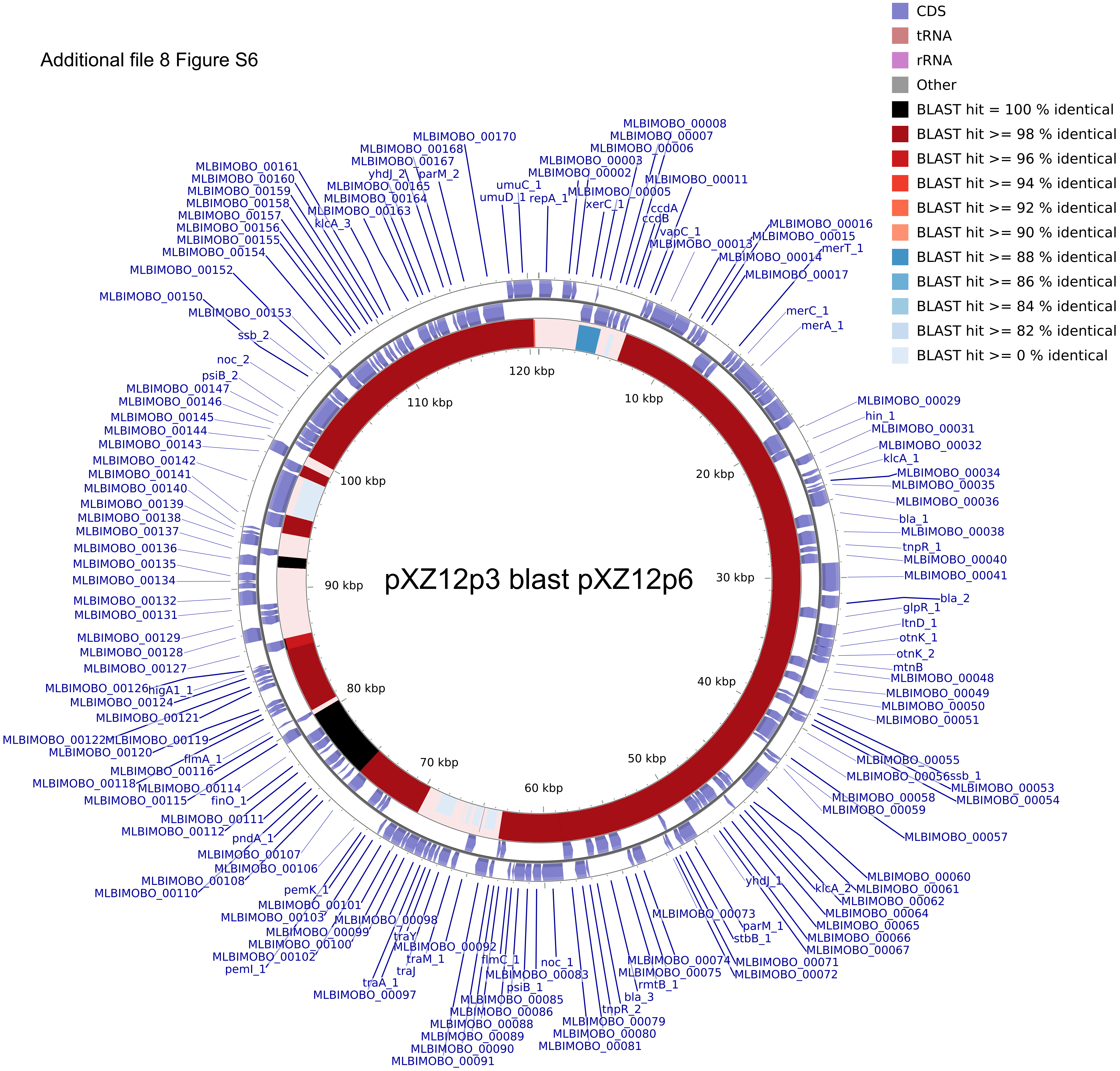

Additional file 9 Figure S7

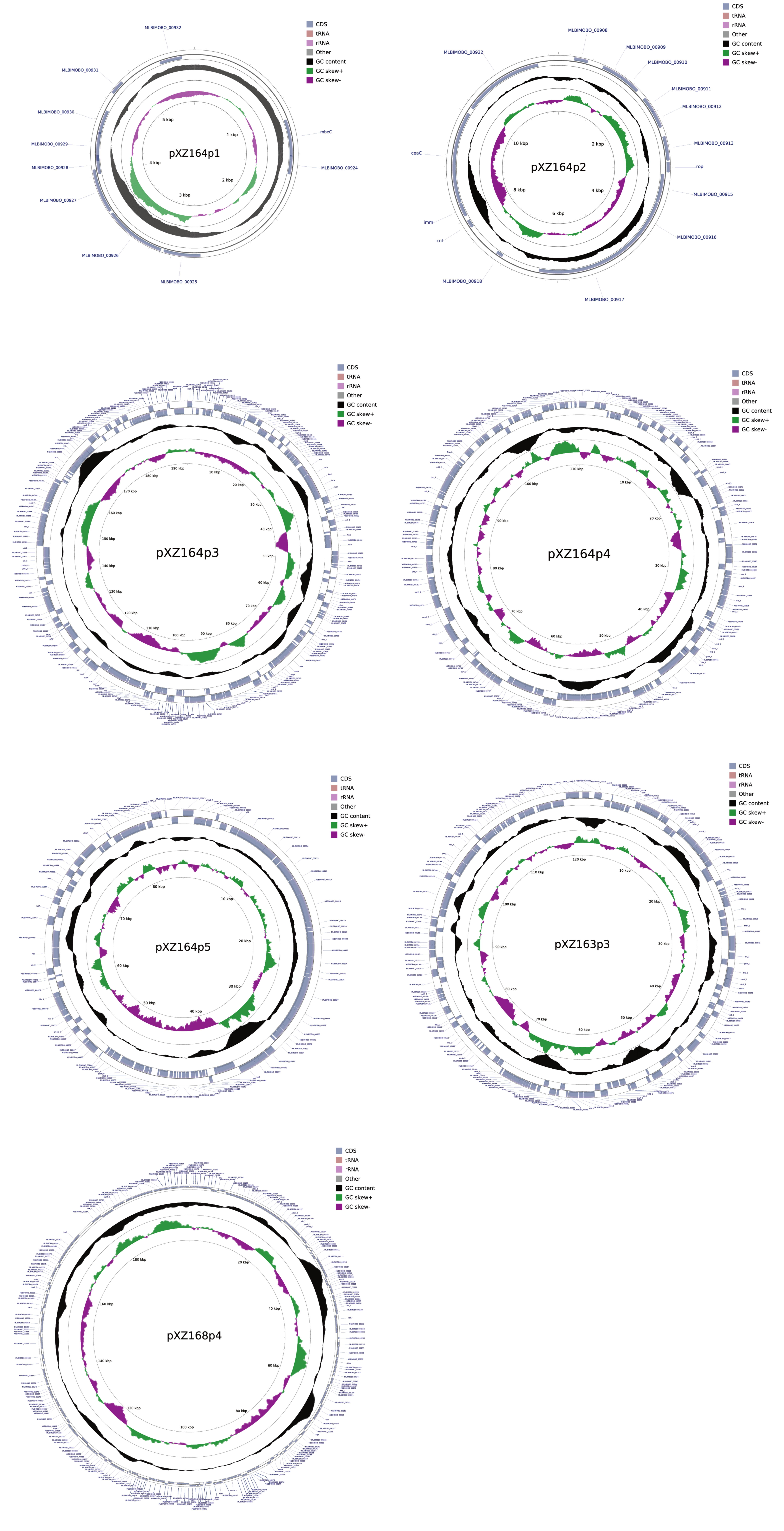

Supplement: Supplemental file 1 — Supplemental material. Download spectrum.01919-22-s0001.pdf, PDF file, 5.5 MB [file spectrum.01919-22-s0001.pdf]
